# Factors affecting 90-day mortality in community and hospital acquired pneumonia patients with or without acute kidney injury

**DOI:** 10.4314/ahs.v22i3.61

**Published:** 2022-09

**Authors:** Derya Hoşgün, Semih Aydemir

**Affiliations:** 1 Atatürk Chest Diseases and Chest Surgery Education and Research Hospital, Department of Intensive Care Unit, Ankara, Turkey. (Chest Disease Specialist , İntensive Care Specialist); 2 Atatürk Chest Diseases and Chest Surgery Education and Research Hospital, Department of Intensive Care Unit, Ankara, Turkey. (Anaesthesiology and Reanimation Specialist)

**Keywords:** Community Acquired Pneumonia, Hospital Acguired Pneumonia, Acute Kidney İnjury

## Abstract

**Background:**

AKI is a significant risk factor for mortality. Inflammatory markers are commonly used in the prediction of prognosis in pneumonia patients. The present study aimed to evaluate the prevalence of AKI in hospitalized CAP and HAP patients and to investigate the role of inexpensive, practical, routinely measured serum biomarkers in predicting 90-day mortality.

**Materials and Methods:**

The retrospective study included 381 patients in CAP patients and HAP patients who were hospitalized in our Chest Diseases clinic or ICU.

**Results:**

Ninety-day mortality occurred in 115 (30.2%) patients (CAP, 28.7%; HAP, 34.7%). AKI was detected in 25.5% of the patients. On multivariate logistic regression analysis, the 90-day mortality risk was 0.931, 1.05, 0.607, and 1.999 times greater in patients with an increased APACHE II score and increased WBC, 1-h creatinine, and 48-h creatinine levels, respectively. In CAP patients, the 90-day mortality risk was 0.296, 0.539, and 1.966 times greater in patients with an increased CURB-65 score and elevated 1-h and 48-h creatinine levels, respectively. In HAP patients, however, the 90-day mortality risk was 3.554 times greater in patients with an increased 48-h creatinine level.

**Conclusion:**

Novel practical scoring systems based on serum creatinine levels are needed for the prediction of long-term prognosis in pneumonia patients.

## Introduction

Community Acguired Pneumonia (CAP) is basically defined as a pneumonia acquired in the community during daily life activities and hospital Acquired Pneumonia (HAP) is defined as a pneumonia that occurs within 48 h after hospital admission or within 48 h after discharge.^1^–^3^ Acute kidney injury (AKI) is a significant factor contributing to mortality in hospitalized pneumonia patients, particularly in patients hospitalized in intensive care unit (ICU).^4^

Pneumonias are often diagnosed based with the aid of biomarkers that are specific to the lung infection or are expressed in response to the inflammation. Although the specificity and sensitivity of these biomarkers may vary, they have high positive predictive values (PPVs) and negative predictive values (NPVs). In some studies, however, CRP has been shown to be an inadequate biomarker in the prediction of prognosis since it is elevated in the settings of stress, extrapulmonary infections, and auto-immunity.^5^,^6^ In contrast, another study reported that PCT is highly useful in predicting the requirement of ICU and vasopressor support.^6^ On the other hand, Neeser et al. evaluated CAP caused by Mycoplasma pneumoniae (M. pneumoniae)and reported that the CRP/PCT ratio (at a cut-off level of 400 mg/µg) could be used for the diagnosis. 7 Recent studies have investigated the efficacy of inexpensive and routinely used biomarkers in the prediction of short- and long-term mortality and prognosis in inflammatory diseases. The studies concluded that the red blood cell distribution width (RDW) values increased despite the variation in the cut-off values and also serum albumin (ALB) levels decreased. Based on these findings, the authors suggested that these two parameters could be used in predicting mortality.^7^–^9^ In a related manner, the RDW/platelet (PLT) ratio has been found to be a prognostic factor in patients with acute pancreatitis and to be an early prognostic factor in patients with severe burn injury.^8^,^9^

The present study was designed to evaluate the prevalence of AKI in hospitalized CAP and HAP patients and to investigate the role of inexpensive, practical, routinely measured serum biomarkers in predicting 90-day mortality.

## Materials And Methods

The retrospective study included a total 381 patients comprising CAP patients and HAP patients who were hospitalized in our Chest Diseases clinic or intensive care unit (ICU) between January 2014 and April 2020. No VAP patients were included in the study. The CAP patients were diagnosed and hospitalized within 24 h after presenting to the emergency service and the HAP patients were diagnosed and hospitalized 48 h after hospital discharge. All the patients were followed up for mortality for 90 days. All the diagnostic and treatment procedures were performed in accordance with international guidelines.^1^–^3^ The study was initiated after obtaining an ethical approval.

Microbiological samples were obtained within the first 72 h of hospitalization and were evaluated by an experienced infectious diseases specialist.CAP patients were grouped based on the Confusion Urea Respiratory Rate Blood Pressure-65 (CURB-65) scoring system. All the patients underwent chest radiography within the first 24 h after the diagnosis and the electronic images showing lobar/multilobar consolidation and ground-glass opacity were retrospectively evaluated by a Chest Diseases specialist. The patients that were hospitalized in ICU were chosen among those who met the 2007 Infectious Disease Society of America (IDSA) and American Thoracic Society (ATS) criteria regarding ICU admission.1 In ICU patients, the Acute Physiology and Chronic Evaluation (APACHE II) score was calculated within the first 24 h of admission for the prediction of mortality and prognosis.^10^ AKI was diagnosed based on the 2012 Kidney Disease Improving Global Outcomes (KDIGO) criteria for staging the severity of AKI (>0.3 mg/dl increase in serum creatinine level within 48 h after admission).^11^

Exclusion criteria were as follows: pregnancy, age below 18 years, presence of an active infection other than pneumonia, VAP, connective tissue disease, prior ICU hospitalization within the last three months, ongoing RRT due to chronic kidney disease (CKD), obstructive uropathy, AKI associated with contrast material and drug-related AKI, history of iron, vitamin B12, or folic acid replacement within the last three months, malignancies, and hematological diseases.

In all patients, WBC count and hemoglobin (HGB), CRP, PCT, PLT, albumin (ALB), and blood urea nitrogen (BUN)levels were measured within the first 24 h after admission and creatinine levels were measured at the 1stand 48thh after admission. Additionally, the RDW/PLT, RDW/ALB, CRP/PCT, and PCT/ALB ratios were calculated for each patient. WBC, HGB, PLT, and RDW were measured using a Mindary BC-6800 automated hematology analyzer with the photometric method and the normal reference ranges accepted for these parameters were 4.6–10.2 10^3/µL,12–16 g/dl, 142–42410^3/µL, and 11.6–17.2%, respectively. CRP, BUN, creatinine, and ALB levels were measured using a Beckman Coulter autoanalyzer with the turbidimetric assay and the normal reference ranges accepted for these parameters were 0–5 mg/L, 8–20 mg/dL, 0.81–1.44 mg/dL, and 3.5–7.2 mg/L. PCT levels were measured on a Siemens ADVIA Centaur XPT Immunoassay System device using the immunoassay test and a PCT level<0.5 ng/ml was accepted to indicate a low risk and a PCT level>2 ng/ml was accepted to indicate a high risk.

## Statistical Analysis

Data were analyzed using SPSS 23.0 (IBM Corp. Released 2015. IBM SPSS Statistics for Windows, Version 23.0. Armonk, NY: IBM Corp.). Categorical variables were expressed as frequencies (n) and percentages (%) and continuous variables were expressed as mean ± standard deviation (SD). Categorical variables were compared using Pearson's Chi-squared test. Multivariate analysis was performed using Logistic Regression Analysis. Normal distribution of data was analyzed using Shapiro-Wilk test. In normally distributed data, Student's t-test was used for comparing two variables and One-Way ANOVA was used for comparing more than two variables. In nonnormally distributed data, Mann-Whitney U test was used for comparing two variables and Kruskal-Wallis test was used for comparing more than two variables. A p value of <0.05 was considered significant.

## Results

The 381 patients comprised 246 (64.6%) men and 135 (35.4%) women with a mean age of 71.64±15.22 years. Ninety-day mortality occurred in 115 (30.2%) patients. Vasopressor/inotropic supprt was provided in 211 (55.4%) and RRT was administered in 47 (12.3%) patients (Table 1).

**Table 1:** Demographic and clinical characteristics

Age	Mean±SD 71.64±15.22	Min-Max 20–97	N 381
**Variables**		N	%
Gender	Male Female	246 135	64.6 35.4
Pneumonia type	CAP HAP	289 92	75.9 24.1
Comorbidities	No Yes	102 279	26.8 73.2
Comorbidity (COPD)	No Yes	228 153	59.8 40.2
Comorbidity (DM)	No Yes	327 54	85.8 14.2
Comorbidity (CAD)	No Yes	232 149	60.9 39.1
Comorbidity (CHF)	No Yes	271 110	71.1 28.9
90-day mortality	No Yes	266 115	69.8 30.2
Vasopressor/inotropic support	Dopamine + noradrenaline Dopamine Noradrenaline None	82 6 123 170	21.5 1.6 32.3 44.6
RRT	IHD CRTT None	16 31 334	4.2 8.1 87.7
Hospitalization setting	ICU Chest diseases clinic	289 92	75.9 24.1
CURB-65 score (in CAP group only)	2 3 4 5	34 36 138 81	11.7 12.3 47.3 27.7

SD : Standard deviation, Min: Minimum, Max: Maximum, ICU: Intensive care unit, APACHE II: Acute Physiology and Chronic Health Evaluation II, CAP: Community-acquired pneumonia, HAP: Hospital-acquired pneumonia, COPD: Chronic obstructive pulmonary disease, DM: Diabetes mellitus, CAD: Coronary artery disease, CHF: Congestive heart failure, IHD: Intermittent hemodialysis, RTT: Renal replacement therapy, CRTT: Continuous renal replacement therapy, CURB-65: Confusion Urea Respiratory Rate Blood Pressure-65

The rate of ICU hospitalization was significantly higher in HAP patients compared to CAP patients (p<0.05). No significant difference was found between the two groups with regard to gender distribution, 90-day mortality, RRT use, and APACHE II score of ICU patients (p>0.05 for all). The requirement of vasopressor/inotropic support was significantly higher in CAP patients compared to HAP patients (p<0.05) (Table 2).

**Table 2:** Demographic and clinical characteristics in each group

Variables		CAP (n=289)	HAP (n=92)	Total (n=381)	*p*
		n (%)	n (%)	n (%)	
Gender	Male	185 (64.0)	61 (66.3)	246 (64.6)	0.689
	Female	104 (36.0)	31 (33.7)	135 (35.4)	
90-day mortality	Yes	83 (28.7)	32 (34.8)	115 (30.2)	0.270
	No	206 (71.3)	60 (65.2)	266 (69.8)	
Hospitalization setting	ICU	188 (65.1)	92(100.0	280 (73.5)	0.000*
	Chest diseases clinic	101 (34.9)	0 (0.0)	101 (26.5)	
Vasopressor / inotropic support	Dopamine + noradrenalin	66 (22.8)	16 (17.4)	82 (21.5)	0.000*
	Dopamine	6 (2.1)	0 (0.0)	6 (1.6)	
	Noradrenalin	73 (25.3)	50 (54.3)	123 (32.3)	
	None	144 (49.8)	26 (28.3)	170 (44.6)	
RRT	IHD	10 (3.5)	6 (6.5)	16 (4.2)	0.370
	CRTT	25 (8.7)	6 (6.5)	31 (8.1)	
	None	254 (87.9)	80 (87.0)	334 (87.7)	
		**TKP** **(n=289)**	**HAP** **(n=92)**	**Total** **(n=381)**	***p***
**Variables**		**Mean±SD**	**Mean±SD**	**Mean±SD**	
Age		70.84±15.86	74.17±12.75	71.64±15.22	0.068
APACHE II score (ICU)		26.60±6.97	27.88±7.41	27.02±7.13	0.162

SD : Standard deviation, ICU : Intensive care unit, APACHE II: Acute Phy siology and Chron ic H ealth Evaluation II, CAP : Community-acquired pneumonia, HAP: Hospital-acquired pneumonia, IHD: Intermittent hemodialysis, RTT: Renal replacement therapy, CRTT: Continuous renal replacement therapy

The mean levels of WBC, RDW, ALB and the mean PCT/ALB ratio were significantly lower and the mean CRP level was significantly higher in CAP patients compared to HAP patients (p<0.05). Nevertheless, no significant difference was found between the two groups with regard to the mean HGB, PCT, PLT, and 48-h creatinine levels and the mean RDW/ALB, RDW/PLT, and CRP/PCT ratios (p>0.05) (Table 3).

**Table 3:** Laboratory findings

	CAP (n=289)	HAP (n=92)	Total (n=381)	*p*
Variables	Mean±SD	Mean±SD	Mean±SD	
WBC (10^3^/µL)	12.75±6.71	14.82±6.14	13.25±6.63	0.009*
HGB (g/dL)	12.50±2.06	12.09±2.54	12.40±2.19	0.119
RDW (%)	16.70±3.54	18.48±2.62	17.13±3.42	0.000*
CRP (mg/L)	69.38±89.34	12.07±8.74	55.54±81.67	0.000*
PCT (ng/ml)	5.69±13.15	7.32±20.40	6.12±15.38	0.386
PLT (103/µL)	246.55±126.4	240.67±108.24	245.13±122.15	0.688
ALB (mg/L)	2.91±0.58	3.08±0.54	2.95±0.58	0.011*
BUN (mg/dL)	43.63±41.63	32.83±27.12	41.02±38.87	0.020*
Creatinine (1-h) (mg/dL)	1.38±1.03	0.97±0.56	1.28±0.96	0.000*
Creatinine (48-h) (mg/dL)	1.07±0.96	1.01±0.53	1.06±0.88	0.548
RDW/ALB ratio	5.98±1.79	6.21±1.67	6.04±1.76	0.289
RDW/PLT ratio	0.10±0.12	0.10±0.09	0.10±0.011	0.864
PCT/ALB ratio	2.26±5.37	2.71±7.97	2.37±6.15	0.012*
CRP/PCT ratio	32.57±10.82	32.42±53.41	24.83±95.84	0.547

SD: Standard deviation, CAP: Community-acquired pneumonia, HAP: Hospital-acquired pneumonia, WBC: White blood cells, HGB: Hemoglobin, RDW: Red blood cell distribution width, CRP: C-reactive protein, PCT: Procalcitonin, PLT: Platelet, ALB: Albumin, BUN: Blood urea nitrogen

Table 4 presents AKI, culture growth, and radiographic localization and findings of infection.

**Table 4:** Acute kidney injury, culture growth, and radiographic localization and findings of infection

Variables		CAP (n=289)	HAP (n=92)	Total (n=381)	p*
		n (%)	n (%)	n (%)	
AKI (KDIGO criteria)	Yes	69 (23.9)	28 (30.4)	97 (25.5)	0.208
	No	220 (76.1)	64 (69.6)	284 (74.5)	
Culture growth	None	229 (79.2)	0 (0.0)	229 (60.1)	0.000*
	Pseudomonas aeruginosa	26 (9.0)	31 (33.7)	57 (15.0)	
	Klebsiella pneumoniae	4 (1.4)	17 (18.5)	21 (5.5)	
	Gr (-) bacilli	10 (3.5)	23 (25.0)	33 (8.7)	
	S. Aerus	2 (0.7)	0 (0.0)	2 (0.5)	
	E.coli	5 (1.7)	0 (0.0)	5 (1.3)	
	Influenza virus	6 (2.1)	0 (0.0)	6 (1.6)	
	Acinetobacter baumannii	7 (2.4)	21 (22.8)	28 (7.3)	
Culture growth (Total)	No	229 (79.2)	0 (0.0)	229 (60.1)	0.000*
	Yes	60 (20.8)	92 (100.0)	152 (39.9)	
Radiographic localization	Lobar	166 (57.4)	38 (41.3)	204 (53.5)	0.007*
	Multilobar	123 (42.6)	54 (58.7)	177 (46.5)	
Radiographic finding	Consolidation	157 (54.3)	31 (33.7)	188 (49.3)	0.000*
	Ground-glass opacity	74 (25.6)	11 (12.0)	85 (22.3)	
	Consolidation + ground-glass opacity	58 (20.1)	50 (54.3)	108 (28.3)	

CAP: Community-acquired pneumonia, HAP: Hospital-acquired pneumonia, AKI: Acute kidney injury, KDIGO: Kidney Disease Improving Global Outcomes, S. Aerus: Staphylococcus aureus, Gr (-): Gram-negative, E. coli: Escherichia coli

No significant difference was found between patients with and without AKI with regard to the type of pneumonia, radiographic localization and findings of the infection, WBC and RDW levels, and the RDW/ALB and CRP/PCT ratios (p>0.05 for all). However, the APACHE II score, CRP, PCT, 1-h and 48-h creatinine levels and the RDW/PLT and PCT/ALB ratios were significantly higher in patients with AKI compared to those without AKI (p<0.05 for all). On multivariate logistic regression analysis, mode of hospitalization (inpatient clinic or ICU), administration of vasopressor/inotropic support with noradrenaline, and an increased 48-h creatinine level were found to be independent risk factors for AKI. Moreover, the mean PLT and ALB levels were significantly higher in AKI patients compared to non-AKI patients (p<0.05). Ninety-day mortality was significantly higher in non-AKI patients compared to AKI patients (p<0.05). However, no significant relationship was found between 90-day mortality and microbiological culture results and the radiographic localization and findings of the infection(p>0.05). Ninety-day mortality was higher in patients receiving intermittent hemodialysis (IHD)compared to those who were not receiving IHD (p<0.05). Similarly, it was significantly higher in patients that were receiving continuous renal replacement therapy (CRRT) and did not have mortality as an outcome compared to other patients (p<0.05 for both). On multivariate logistic regression analysis, the 90-day mortality risk was 0.931,1.05, 0.607, and 1.999 times greater in patients with an increased APACHE II score and increased WBC, 1-h creatinine, and 48-h creatinine levels, respectively. In CAP patients, the 90-day mortality risk was 0.296, 0.539, and 1.966 times greater in patients with an increased CURB-65 score and elevated 1-h and 48-h creatinine levels, respectively (Table 5).

**Table 5:** Risk factors for 90-day mortality in CAP patients

Variables	OR	OR (95% CI)		*p* *
		Lower	Upper	
CURB-65 score	0.296	0.174	0.503	0.000*
WBC (10^3^/µL)	1.042	0.988	1.099	0.127
HGB (g/dL)	0.895	0.769	1.041	0.151
RDW (%)	0.943	0.719	1.237	0.673
CRP (mg/L)	0.999	0.996	1.002	0.594
PCT (ng/ml)	0.949	0.845	1.066	0.376
PLT (103/µL)	1.000	0.997	1.003	0.941
ALB (mg/L)	1.147	0.201	6.558	0.878
Creatinine (1-h) (mg/dL)	0.539	0.367	0.790	**0.002** *
Creatinine (48-h) (mg/dL)	1.966	1.323	2.922	**0.001** *
RDW/PLT ratio	0.581	0.039	8.650	0.693
RDW/ALB ratio	0.967	0.472	1.981	0.926
CRP/PCT ratio	1.000	1.000	1.000	0.490
PCT/ALB ratio	1.117	0.841	1.484	0.446

* *p*<0.05

CAP: Community-acquired pneumonia, OR: Odds ratio, CI: Confidence Interval, CURB-65: Confusion Urea Respiratory Rate Blood Pressure-65, WBC: White blood cells, HGB: Hemoglobin, RDW: Red blood cell distribution width, CRP: C-reactive protein, PCT: Procalcitonin, PLT: Platelet, ALB: Albumin

In HAP patients, however, the 90-day mortality risk was 3.554 times greater in patients with an increased 48-h creatinine level (Table 6).

**Table 6:** Risk factors for 90-day mortality in HAP patients

Variables	OR	OR (95% CI)		*p* *
		Lower	Upper	
WBC (10^3^/µL)	1.115	0.933	1.252	0.066
HGB (g/dL)	0.961	0.762	1.213	0.741
RDW (%)	1.311	0.738	2.330	0.356
CRP (mg/L)	0.937	0.875	1.003	0.062
PCT (ng/ml)	1.075	0.781	1.479	0.658
PLT (10^3^/µL)	1.003	0.995	1.011	0.511
ALB (mg/L)	0.200	0.008	4.874	0.323
Creatinine (1-h) (mg/dL)	1.551	0.509	4.727	0.440
Creatinine (48-h) (mg/dL)	3.554	1.015	12.449	**0.047** *
RDW/PLT ratio	4.580	0.153	2.458	0.470
RDW/ALB ratio	0.568	0.137	2.357	0.436
CRP/PCT ratio	1.007	0.995	1.018	0.249
PCT/ALB ratio	0.854	0.355	2.051	0.724

* p<0.05

HAP: Hospital-acquired pneumonia, OR: Odds ratio, CI: Confidence Interval, WBC: White blood cells, HGB: Hemoglobin, RDW: Red blood cell distribution width, CRP: C-reactive protein, PCT: Procalcitonin, PLT: Platelet, ALB: Albumin

## Discussion

The mortality rate in CAP patients has been shown to be 8.2–30% and to be 25–50% in patients requiring hospitalization.^12^ HAP is a frequently seen hospital-acquired infection, with a reported mortality rate of 25–50%.^2^,^13^ CURB-65 and the Pneumonia Severity Index (PSI) are commonly used in CAP patients, particularly in the prediction of prognosis and mortality and also in the decision-making processes related to hospitalization and ICU requirement .APACHE II score is a frequently used scoring system in the prediction of mortality and disease severity in CAP and HAP patients hospitalized in ICU.14 Studies have shown that the CAP patients with a CURB-65 score of ≥3 areat 22% greater risk of mortality and the pneumonia patients that are hospitalized in ICU and have an APACHE II score of >24 have a greater risk of mortality.^14^ In the present study, 90-day mortality rate was significantly higher in HAP patients (34.7%) compared to CAP patients (28.7%) (p=0.270). These rates were consistent with those reported in the literature.13 Recent studies have demonstrated that the mortality rates in CAP and HAP patients have become highly similar due to the fact that the microbiological agents associated with HAP have been detected in CAP patients as well and there has been a significant increase in the number of newly diagnosed pneumonia cases owing to the advancements in age-related healthcare services.^1^–^3^

In our study, no significant difference was found between CAP and HAP patients with regard to mortality rate, which could be attributed to the increased CURB-65 scores in CAP patients (range, 4–5), the increased ICU requirement in CAP patients (65.05%), the growth of similar microbiological agents in both groups, and the exclusion of VAP patients. On the other hand, the increased CURB-65 scores in CAP patients were found to be an independent risk factor, which was found to increase the mortality risk by 0.296 times. Studies investigating the relationship between CURB-65 score and mortality have mostly focused on short-term mortality and thus there are very few studies reporting on long-term mortality. In studies evaluating CAP patients, initial disease severity, age, cardiovascular comorbidities, and inflammation have been shown to be predictive factors for long-term mortality.^15^ To our knowledge, this study is one of the rarest studies in the literature to show the efficacy ofCURB-65 score in predicting long-term mortality in CAP patients. Additionally, the study also aimed to improve this efficacy by adding inexpensive, practical, and routinely measured inflammatory biomarkers into the analysis.

The analysis revealed that the 90-day mortality risk was 0.931 times greater in patients that were hospitalized in ICU and had an increased APACHE II score. The APACHE II score is commonly used in the prediction of early in-hospital mortality in critically ill patients . In our study, the APACHE II scores were clculated only within the first 24 h of ICU admission and no additional calculations were performed, unlike in many studies in the literature .Hosseini et al. found the APACHE II score as a significant predictor of 6-month mortality in surgical and medical ICU patients.^16^ Although our study had similar outcomes with the studies in the literature regarding the role of the APACHE II score in predicting mortality in pneumonia patients, the APACHE II scores of our patients were not further analyzed by Multivariate Logistic Regression analysis due to the small numbers of ICU patients in our CAP and HAP groups.

Acute kidney injury (AKI) is defined as the sudden loss of kidney function resulting in the inept accumulation of waste products such as urea and nitrogenous waste products.^17^ Bagai et al. evaluated 122 patients with CAP and detected AKI in 40.2% of the patients who had a higher risk of in-hospital mortality.18 A multicentric study evaluated 11,500,546 pneumonia hospitalizations and reported that RRT was administered in 4.3% and AKI-related mortality occurred in 32.4% of the patients.^19^ Another study evaluated sepsis patients and detected AKI in more than 50% of the patients.^20^ In the present study, a diagnosis AKI was established based on based on the KDIGO criteria and AKI was diagnosed in 25.5% of the patients, which was consistent with those reported in the studies investigating CAP and HAP patients.^18^,^19^ Moreover, RRT was administered in 12.3% of our AKI patients. Literature indicates that RRT is used in 12–25% of sepsis patients.^20^,^21^ Accordingly, the rate of RRT use in our study was consistent with those reported in the literature. Contrariwise, no significant relationship was found between RRT use and AKI (p=0.370 and p=0.208, respectively), which could be explained by the fact that both CAP and HAP patients had severe pneumonia and had increased requirement of vasopressor/inotropic support and all the AKI patients were hospitalized in ICU.18 Due to the retrospective nature of our study, no data regarding the factors associated with RRT such as body mass index (BMI), early or late initiation of RRT, administration of CRRT, and the dosing and duration of IDH could be attained and thus no standardization could be performed in the study.^20^,^21^ This limitation could be associated with the higher mortality rate in our non-AKI patients. In line with the literature, mode of hospitalization (inpatient clinic or ICU), administration of vasopressor/inotropic support with noradrenaline, and an increased 48-h creatinine level were found to be independent risk factors for AKI (p<0.05 for all)^16^.

In kidney function tests, BUN levels typically increase in the setting of dehydration, and serum creatinine level is affected not only by kidney dysfunction but also by age, gender, nutritional status, liver disease, and fluid volume status . Cho et al. found that increased creatinine levels were a useful predictor of short-term mortality in patients with sepsis-induced AKI that were receiving RRT.^22^ In our study, serum BUN levels were significantly higher in CAP patients compared to HAP patients, which could be associated with the higher prevalence of dehydration findings and the community origin of the infection in CAP patients. Moreover, the higher BUN levels in CAP patients were found to result in higher 1-h creatinine levels in these patients compared to those in HAP patients. In our study, the 1-h and 48-h creatinine levels in CAP patients, as consistent with the literature, were found to be significant predictors of mortality. In contrast, only the 48-h creatinine level was found to be a significant predictor of mortality in HAP patients, which could be attributed to the hospital origin of the infection, the lower prevalence of dehydration findings in CAP patients, and the exacerbation of the disease within the first 72 h of hospitalization. Our study had a limitation regarding the creatinine levels. Due to the retrospective nature of our study, no data regarding patients' nutritional status, BMI, and fluid volume status could be attained and thus no standardization could be performed for the medical therapies performed in the study.

A previous study evaluated pneumonia patients that were hospitalized in the clinic or ICU and reported that serial measurement of CRP and PCT levels within the first 72 h of hospitalization was highly useful in predicting treatment response, prognosis, and mortality.^5^,^7^,^23^ Albumin is secreted from liver cells in response to oxidative stress in the setting of inflammation and has been shown to be a prognostic factor in predicting mortality in pneumonia patients.^23^,^24^ RDW, WBC, and PLT are routinely measured blood parameters that have been shown to increase or decrease in the setting of infection.^26^ Recent studies have investigated the diagnostic role of the ratios of these parameters to one another due to the fact that these parameters have been shown to increase or decrease in the setting of iflammation and their specificity and sensitivity levels are different from one another. A previous study evaluated CAP caused by M. pneumoniae and reported that the increased CRP/PCT ratio calculated on admission could have a diagnostic value.^7^–^9^ Luo et al. evaluated 140 patients with urosepsis and demonstrated that the PCT/ALB ratio could be useful in the early diagnosis and clinical practice.^27^ Yoo et al. reported that the RDW/ALB ratio provided better outcomes in predicting 60-day mortality compared to RDW alone in patients with acute respiratory distress syndrome (ARDS).^28^ In a previous retrospective study, Wang et al. evaluated children with sepsis and reported that the RDW/PLT ratio calculated on admission was closely associated with the prognosis.^29^,^30^

In our study, the WBC, RDW, ALB levels and the PCT/ALB ratio were significantly lower and the CRP level was significantly higher in CAP patients compared to HAP patients, and ALB, CRP, and the PCT/ALB ratio were found to be predictors of disease severity in CAP. Moreover, CAP and HAP patients had similar mortality rates and the CAP patients that received outpatient care and VAP patients were excluded from the study, which could have affected the laboratory findings obtained in the study. On the other hand, the RDW/PLT, RDW/ALB, CRP/PCT, and PCT/ALB ratios, which have been investigated in recent studies, had no significant effect in the prediction of 90-day mortality. Similarly, serum creatinine level had no significant effect in the prediction of 90-day mortality, which could be associated with the high rates of AKI and RRT use in our patients, which are indicators of disease severity.

## Conclusion

The results implicated that there is need for novel scoring systems similar to CURB-65 score, which is used for CAP patients, for the prediction of prognosis and disease severity in HAP patients. Additionally, to evaluate the role of CURB-65 in predicting long-trm mortality in CAP patients, novel scoring systems that are based on serum creatinine levels rather than serum BUN levels are needed. Further multicentric prospective studies performing repeated measurements of routinely measured inflammatory biomarkers are needed to investigate the role of these biomarkers in the prediction of disease severity and prognosis in pneumonia patients.
